# Human immunodeficiency virus and human papilloma virus - why HPV-induced lesions do not spontaneously resolve and why therapeutic vaccination can be successful

**DOI:** 10.1186/1479-5876-7-108

**Published:** 2009-12-18

**Authors:** Sjoerd H van der Burg, Joel M Palefsky

**Affiliations:** 1 Department of Clinical Oncology, Leiden University Medical Center, Leiden, The Netherlands; 2 Department of Medicine, University of California, San Francisco, San Francisco, CA, USA

## Abstract

HIV and HPV can both cause chronic infections and are acquired during sexual contact. HIV infection results in a progressive loss of CD4+ T cells that is associated with an increased prevalence of HPV infections, type-specific persistence and an increase in HPV-associated malignancies. On the one hand this illustrates the important role of HPV-specific CD4+ helper T-cell immunity, on the other it shows the Achilles heel of the HPV-specific immune response. The use of highly active antiretroviral therapy (HAART) results in a rapid reduction of HIV and a reconstitution of systemic CD4+ T-cell levels. The use of HAART thus has the potential to raise immunity to HPV but to the surprise of many, the incidence of HPV-induced diseases has increased rather than declined since the introduction of HAART. Here, the knowledge on how HPV-induced diseases develop in the face of a non-compromised immune system will be used to explain why the effect of HAART on HPV-induced diseases is modest at best. Furthermore, exciting new data in the field of therapeutic vaccines against HPV will be discussed as this may form a more durable and clinically successful therapeutic approach for the treatment of HPV-induced high-grade lesions in HIV-positive subjects on HAART.

## Introduction

Human papilloma virus (HPV) is the most commonly sexually transmitted agent worldwide. A high prevalence of HPV has been reported especially among young sexually active individuals. Persistent infection with oncogenic HPV types, in particular HPV16, are causally related to the development of anogenital lesions like cervical intra-epithelial neoplasia (CIN), vulvar intraepithelial neoplasia (VIN) and anal intraepithelial neoplasia (AIN) as well as their subsequent progression to invasive squamous cell carcinoma [1-5]. While HPV infection is asymptomatic in the great majority of immunocompetent individuals, a small proportion of men and women fail to control viral infection and develop HPV-related malignancies.

The incidence of cervical as well as anal precursor lesions and cancer is markedly higher in HIV-positive men and women [6-8] compared with HIV-negative men and women. In men who have sex with men (MSM) the incidence of AIN and anal cancer was already particularly high and incidence rates are substantially elevated in the population of HIV-positive MSM. Furthermore, the risk of other HPV-associated cancers of the oropharynx, penis, vagina, and vulva is also increased among persons with AIDS [9]. The introduction of antiretroviral therapy in the mid-nineties restored immune responses to several AIDS-defining opportunistic infectious agents such as cytomegalovirus and human herpes virus-8, as well as significantly changed the prognosis and mortality rates of HIV-infected subjects. However, the longer survival of highly active antiretroviral therapy (HAART)-treated subjects led to a high incidence and steady increase in HPV-related malignancies both in women and men [7-11]. This counter-intuitive observation of detecting more HPV-induced malignancies after restoration of the immune system requires an explanation, and the answer may be found in studies on HPV-specific immunity in immune competent individuals.

## CD4+ T cells, the Achilles heel of HPV-specific immunity

Epidemiological studies have shown a strikingly high prevalence of oncogenic HPV types in the general population [12-15]. For example the cumulative lifetime incidence of HPV16 infection is estimated to be over 50% [16]. In most healthy persons the immune system succeeds in eliminating oncogenic HPV types before malignancies develop [13,17]. Natural history studies [18] show that most (90%) low-grade cervical intraepithelial neoplasia (CIN 1) can regress spontaneously and this is attributed to the fact that many CIN 1 lesions are induced by the low-risk non-oncogenic HPV types as well as the development of HPV antigen-specific cellular immune responses. At the time of spontaneous regression of HPV-infected genital warts, the lesions are infiltrated with CD8+ cytotoxic T-cells (CTL), CD4^+ ^T-cells and macrophages [19]. This observation fits well with the general notion that the protective immune response to chronic viral infections is a polyfunctional type 1 response of both CD4+ helper T cells and CD8+ cytotoxic T cells [20-24]. In addition to their direct effects, virus-specific CD4+ T cells are needed to sustain CD8+ effector T-cell responses as well as to activate innate effector cells. Comprehensive studies on HPV16-specific immunity have revealed that HPV16-specific CD4+ T-cell responses and HPV16-specific CTL responses directed against the viral early antigens E2, E6 and/or E7 are detected in peripheral blood mononuclear cell (PBMC) cultures of the majority of healthy individuals. In general, HPV-specific CD4+ T-cell immunity comprises both Th1- (IFNγ) and Th2-producing (IL-5) T cells reactive to a broad array of epitopes within these antigens [25-30]. Moreover, these circulating HPV16-specific CD4+ and CD8+ T cells are able to migrate from the circulation to the epithelium upon antigenic challenge in healthy subjects [31]. These observations suggest that successful defense against HPV16 infection is commonly associated with the induction of a systemic effector T-cell response against the viral early antigens. This notion is sustained by our recent observation that the vaccine-induced regression of HPV16-induced high-grade VIN was associated with a strong and broad IFNγ-associated CD4+ T-cell response against E6 and E7 [32].

Unfortunately, HIV infection results in a progressive loss of CD4+ T cells. When one considers the type of immune response needed to deal with chronic viral infections in general, the frequent detection of HPV-specific CD4+ T-cell reactivity in protected healthy individuals as well as the association between HPV-specific CD4+ T cells and regression of HPV-induced premalignant lesions, it becomes clear why the loss of CD4+ T-cells is associated with an increased prevalence of HPV infections, type-specific persistence and an increase of HPV-associated malignancies [33]. On the one hand this illustrates the important role of HPV-specific CD4+ helper T-cell immunity, on the other it shows that HPV-specific CD4+ T-cell immunity is the Achilles heel of protection against HPV-induced disease.

## Restoration of CD4+ T-cell immunity to pathogens requires antigen exposure under inflammatory conditions

HAART consists of an antiretroviral drug regimen which combines inhibitors of the HIV reverse transcriptase and protease. Depending on the HIV disease stage and the level of HIV viral control over time, the use of HAART results in an increase in the number of CD4+ T cells that may reach (near) normal counts within 2-6 years [34]. Initially HAART induces a rapid increase in CD4+ T-cell counts as a result of the redistribution of memory T cells that had been sequestered in inflamed lymphoid tissues that have come to rest as a result of a significant drop of the HIV viral load when HIV is controlled. The second phase is slower and thought to reflect the re-expansion of naïve CD4+ T cells and re-diversification of the T-cell repertoire following the treatment of this chronic infection [34].

The increase in CD4+ T-cell counts coincides with the decrease in several of the AIDS-defining illnesses arising from opportunistic infections and the detection of T-cell responses against agents such as CMV [35], Candida, Mycobacterium and Streptococcus [36,37]. This occurs a few months after therapy has commenced and is thought to arise after exposure to sufficient quantities of the targeted antigens upon (re-)infection with these pathogens. HIV-specific T cells are only detected at the time of a primary infection or when HAART is given before the onset of CD4+ T cell depletion [34] but not in chronically-infected HAART-treated patients [36,37]. However, Jansen et al. observed an increase in HIV-specific CD4+ T-cell proliferative capacity after 55 months of HAART [38]. These data indicate that chronic infection results in the depletion of HIV-specific immunity - which sounds reasonable as CD4+ T cells are a prime target of HIV - and suggest that new thymic emigrants are only stimulated to respond to HIV over time. The difference in the kinetics of the cellular response to opportunistic pathogens and HIV is best explained by a difference in the host's exposure to antigenic stimuli. The rapid decline of HIV - and as such the amount of HIV antigens which can be presented to the immune system - following HAART is likely to result in suboptimal stimulation of the immune system to HIV. This notion is sustained by the observations that HIV-specific central and effector CD4+ and CD8+ memory T cell populations rapidly disappear from the peripheral blood of infected individuals under HAART [39] while re-exposure of the immune system to HIV during structured therapy interruption (STI) after one year of HAART results in expansion of HIV-specific CD4+ and CD8+ T cells [40]. The important point here is that restoration of immunity in HIV-infected patients on HAART to infectious agents requires (re-)infection and exposure to sufficient amounts of pathogen-derived antigens under inflammatory conditions.

## Failure to restore protective HPV-specific immunity in HIV-positive patients on HAART

Thus far, there is no evidence showing that the oncogenic behaviour of HPV is altered by HIV[41] and while HIV-induced immunosuppression can be held accountable for the increased incidence of precursor lesions it does not explain why these lesions do not resolve when HAART is given and immunosuppression is alleviated.

Some previous studies do report a positive effect of HAART on the natural history of HPV-induced pre-malignancies in HIV-infected subjects. A close look at the data shows us that most of the effects noted are among patients with low-grade lesions with response rates of ~35% [42,43], which is still lower than what is observed in immunocompetent individuals of whom ~60% clears a low-grade CIN within 12 months [44]. Notably, 25% of the HIV-infected low-grade CIN subjects on HAART still progress to high-grade CIN [43] and HIV-infected patients with high-grade CIN often do not show regression when treated with HAART [43,45]. One should realize that in many HIV-positive patients on HAART the HPV infections and HPV-induced lesions are not newly acquired but reflect persistence and/or recently reactivated prior infections of the HPV types detected despite an increase in CD4+ T cell levels [8]. In a number of cases it may also reflect new exposure [46]. The persistence of HPV and HPV-induced lesions indicate that HPV-infection was not counteracted in the first place and that the virus was allowed to establish LSIL and/or HSIL lesions before the capacity of the immune system to respond was restored.

So what happens with the HPV-specific immune response in patients on HAART? It is hard to know as there are few studies on the kinetics of HPV-specific immunity during HAART. Fortunately, there is considerable knowledge on HPV-specific immunity in immunocompetent individuals that allows us to understand why HAART cannot simply restore full protective immunity to HPV in HIV-infected subjects.

## Immunity to HPV-induced lesions in immunocompetent individuals

In contrast to the opportunistic pathogens or HIV, HPV is rather a stealthy virus as it causes minimal inflammation, allowing it to persist at detectable levels for 12-18 months in immunocompetent subjects [47]. From a teleologic point of view this is necessary for HPV as it requires the full cycle of keratinocyte differentiation to produce its own viral particles and inflammatory signals may jeopardize its capacity to replicate. Although much work is still needed in this area, HPV seems to alter transcriptional activity of the IFNβ and NFkB-pathways resulting in a decreased ability of keratinocytes to produce the necessary cytokines and chemokines to attract the adaptive immune system [48-50]. The identification of HPV-induced low-grade or high-grade lesions reflects molecular changes in the normal program of epithelial cell differentiation that occur following infection. Importantly, the timely expression of viral gene products and the linked production of viral particles are progressively disturbed during neoplastic progression [51]. In addition, the development of such lesions is associated with a locally altered cytokine environment with an increase in IL-10 and a decrease in proinflammatory cytokines [52-54]. The progression rate of high-grade lesions of the cervix, vulva or anal region to cancer in immunocompetent subjects is similar among the different types of lesions (9-13%) [55-57], and regressions are only occasionally observed.

A comparison of immune presentation of opportunistic pathogens and HPV indicates that there is less inflammation and there are lower amounts of antigens available to the immune system with HPV infection. One could compare the presentation of HPV antigens in immunocompetent subjects to that of HIV antigens in patients on HAART, as in both cases the induction of detectable immune responses may take a while. When HPV-induced lesions develop, the production of viral antigens is severely altered due to the loss of a productive infection. Some viral antigens are not produced anymore (e.g. E2) whereas others may increase in time (e.g. E7). Most importantly antigen-presenting cells (APC) that are present in the local region and whose normal role is to ingest and present the viral antigens to T cells, are functionally altered as these APC are exposed to an immunosuppressive environment and become tolerized [58]. As a consequence the immune response to HPV is different in patients with HPV-induced lesions when compared with healthy individuals who do not have HPV-associated disease (see above).

In a large prospective study on the clinical course of low-grade CIN we have studied HPV16-specific immunity in relation to clinical outcome [59]. HPV16-specific IFNγ-associated T-cell responses were detected in only half of the patients with an HPV16+ low-grade CIN, and responses were predominantly to HPV16 E2 and E6. Interestingly, the presence of HPV16 E2-specific T-cell responses correlated with absence of progression of HPV16+ lesions but this was only a small group [59]. Thus the immune system clearly fails to activate CD4+ IFNγ-producing HPV-specific T cells in half of the immunocompetent patients with low-grade CIN and only in a minority of the subjects the immune response is strong enough to induce regression.

The HPV-specific immune response in patients with high-grade CIN lesions is even worse. The accumulated data from a number of different studies on patients with HPV16+ high-grade CIN revealed that HPV16-specific T-cell responses were absent in the circulation of the majority of patients who visit the clinic for treatment of an HPV16+ high-grade lesion. Notably, the quality of the immune response in those patients who did show HPV16-specific reactivity was low in the sense that most of the detected HPV16-specific T-cell responses did not include secretion of pro-inflammatory cytokines such as IFNγ. In the end, more than 75% of all patients with a high-grade lesion failed to develop an HPV16-specific cellular immune response which would remotely resemble that of what was seen in healthy individuals [26,59-62]. Importantly, HPV16-specific T-cell reactivity was predominantly found in patients returning to the clinic for repetitive treatment of a persistent or recurrent HPV16+ high-grade CIN after initial destructive treatment [61]. This suggests that the induction of HPV-specific reactivity in patients with high-grade CIN requires sufficient exposure to antigen (achieved by persistence/recurrence) as well as inflammation such as is caused by destructive treatment. Unfortunately, this is the case in only a minority of women with high-grade CIN. Moreover, when the viral antigens are presented it is usually in a suppressive environment and as a result a non-beneficial HPV-specific immune response develops that is unable to induce the regression of an HPV-induced lesion. This notion is consistent with our observation that high-grade CIN-infiltrating T-cell cultures can contain HPV16-specific regulatory T-cells [61]. Thus if an HPV-specific immune is present in patients with high-grade CIN it consists of T-cells that do not produce IFNγ and sometimes even has a suppressive signature. This type of immunity is in clear contrast with that found in healthy individuals or patients in whom their lesions regressed [25-27,32,63]. Aforementioned data on HPV16-specific T-cell immunity in HIV-positive patients on HAART are lacking but it would be safe to assume that the response rate and type of HPV-specific immune response in HIV-positive patients on HAART at least is not better than that of immunocompetent patients with low-grade or high-grade lesions.

Current literature indicates that HPV-induced lesions are less likely to regress in immunocompetent [64] or HIV-positive patients [65] when these lesions - being either low-grade or high-grade - are induced by high-risk HPV types as compared to low-risk HPV types. Moreover, the accumulated data on HPV16-specific immunity in immunocompetent patients clearly show that - even when the immune system is not compromised - an established high-risk HPV-induced lesion fails to trigger a functional HPV-specific immune response. Considering that the prevalence of HPV and HPV-associated disease are much higher in HIV-infected men and women [8,66-69], it is highly likely that the HPV-specific immune response in patients on HAART will not be induced in most of them or in some cases may resemble that of non-immunocompromised patients with lesions, i.e., does not confer protective immunity.

It is not fair to expect that HAART would lead to regression of HPV-induced cancer as this also poses a general problem among immunocompetent patients with cancer. Furthermore, cervical cancer is strongly associated with failure to mount a strong HPV-specific type 1 T-helper and cytotoxic T lymphocyte (CTL) response and the induction of HPV-specific regulatory T cells [26,30,70-72]. Furthermore, CD8+ T cells may fail to migrate into the tumor cell nests and when tumors are infiltrated by CD8 T cells it coincides with infiltration by CD4+Foxp3+ regulatory T cells. Moreover, half of the tumor-infiltrating T cells express the programmed cell death receptor 1 as a sign of T-cell exhaustion [73-75]. In addition, the loss of human leukocyte antigens - which presents antigens to the T cells - is often observed and has a clear negative impact on patient survival [74].

## Non-specific treatment is associated with high recurrence rates

Screening and treatment options for CIN and cancer are well established and consequently the incidence of cervical cancer in HIV-positive women has not increased following the implementation of HAART. There is, however, a strong increase in the incidence of anal diseases in both men and women [10]. Although cytological screening for AIN - analogous to cervical screening - has been proposed [76] this is not common practice. Similarly, treatment guidelines for anal lesions are yet not available but the different strategies used so far fall into the categories of topical treatments, ablative treatments and immunotherapy. In this they resemble current treatment options used for the treatment of VIN in immunocompetent patients.

In a recent review of Kreuter et al. [77] an overview on the response rates and recurrence rates associated with different therapies for AIN is presented. In summary, ablative treatments (e.g. surgery, infrared, laser therapy) in general show a high response rate to treatment but also a high recurrence rate (38-79%) within an average of 1-2 years. A few studies indicated that topical treatment of patients with AIN1-AIN3 with imiquimod may result in good clinical responses in patients with good compliance [78-80], albeit that these results have to be confirmed by others. Notably, despite good initial results the recurrence rate of 26-29% is still high [78-80]. If the response of AIN to imiquimod is indeed that good, it resembles that seen for the treatment of vulvar lesions in immunocompetent patients [81] in whom clinical response was related to the presence of weak IFNγ-producing HPV-specific T cells [63]. In view of this association between HPV-immunity and therapy response one may expect that also the responsiveness of HIV-positive AIN patients on HAART to imiquimod is related to the presence of HPV-specific immune responses. This suggests that HPV-specific immunity may also develop in patients with AIN on HAART albeit not sufficiently to induce the regression of the lesion without the help of a local induced of inflammation such as imiquimod. To clarify this issue new studies are needed to measure HPV-specific immunity in HAART-treated patients.

## HPV-specific therapeutic vaccination to treat may now become an option

The clear link between HPV16 and cancers of the cervix, vulva and anal region has prompted the development of two types of vaccines. One type is focused on the prevention of high-risk HPV-infection for which Franceschi and De Vuyst argued that its success to prevent AIN and anal cancer depends on the administration of the vaccine before onset of sexual activity, its protective efficacy in men as well as the willingness to expand vaccine programs to both sexes [82]. The other type is a therapeutic vaccine aiming at strengthening the HPV16-specific T-cell response. In contrast to the natural context in which the immune system is exposed to lesion-derived HPV antigens, therapeutic vaccines can ensure the deliverance of sufficient quantities of HPV antigens in a highly stimulatory context, and as such may be able to restore an adequate HPV-specific immune response able to induce the regression and clearance of HPV-induced lesions. Thus far, two vaccines have been used to treat high-grade AIN in HIV-negative men (ZYC101) or HIV-positive men (SGN-00101). The vaccines were well-tolerated but did not induce clinical responses higher than what would spontaneously occur in these patient populations [83,84]. This result was not specific to AIN, since these vaccines also were unable to induce regression of CIN in immunocompetent patients[85,86].

Recently, a different type of vaccine consisting of overlapping HPV16 E6 and E7 synthetic long peptides (HPV16-SLP), was reported to induce strong and broad CD4+ T-helper and CD8+ CTL responses in >95% of patients with HPV16-induced cervical cancer [87,88]. The reason for its strong immunogenicity has been extensively reviewed [89]. A phase II clinical trial in which patients with VIN3 were treated with HPV16-SLP showed an objective clinical response rate of 79% and complete and durable (>24 months) complete regression of the lesion in 47% of the patients [32]. The spontaneous regression of these lesions is <1.5% [90]. Furthermore, there was a clear correlation with the strength of the IFNγ-producing HPV16-specific T-cell response and clinical outcome [32]. Interestingly, about half of the patients treated as well as half of the patients with a complete regression had multifocal disease some of which extended to the perianal region. Because of the similarities between immunocompetent patients and HIV-positive patients on HAART, in particular their restored immune response to opportunistic infections, these results offer hope for the treatment of HIV-positive patients on HAART. There are, however, still many issues to consider as we don't fully understand HPV-specific immunity in HIV-positive patients on HAART yet. One of these issues is the size of the lesion as in many cases HIV-positive patients have large lesions. The relatively larger VIN3 lesions were less likely to regress in immunocompetent VIN3 patients with HPV16-SLP [32] and this is clearly associated with an altered immune response (van der Burg, unpublished), suggesting that if one would like to have a chance to be successful one should start treatment as soon as patients receive HAART and/or while lesions are limited in size. One could even imagine vaccinating patients showing only the signs of an HPV16 infection with HPV16-SLP if significant reduction in development of HPV16-related disease can be demonstrated in prospective studies.

## Abbreviations

AIN: anal intraepithelial neoplasia; APC: antigen presenting cell; CIN: cervical intra-epithelial neoplasia; CTL: cytotoxic T lymphocyte; HAART: highly active antiretroviral therapy; HIV: Human Immunodeficiency Virus; HPV: Human Papilloma Virus; IFN: interferon; SLP: synthetic long peptides; VIN: vulvar intraepithelial neoplasia.

## Competing interests

SHvdB in an employee of the Leiden University Medical Center (LUMC), which holds a patent on the use of synthetic long peptides as vaccine (US 7,202,034: *Long peptides of 22-45 amino acid residues that induce and/or enhance antigen specific immune responses*). SHvdB is one of the inventors of the patent and reports to serve as a non-paid member of the strategy team and steering committee of ISA Pharmaceuticals, a biotech company which has licensed the patent from the LUMC. SHvdB has not received any payment for speaking, consulting, patents or royalties with respect to the present study.

JMP has not received any payment for speaking, consulting, patents or royalties with respect to the present study.

## Authors' contributions

SHvdB and JMP drafted, read and approved the final manuscript.
